# ANO1 as a marker of oral squamous cell carcinoma and silencing ANO1 suppresses migration of human scc-25 cells

**DOI:** 10.4317/medoral.19076

**Published:** 2013-12-07

**Authors:** Yadong Li, Jinsong Zhang, Suling Hong

**Affiliations:** 1 Department of Oral and Maxillofacial Surgery, the First Affiliated Hospital of Chongqing Medical University, Chongqing 400016, China; 2 Department of Otolaryngology, the First Affiliated Hospital of Chongqing Medical University, Chongqing, 400016, China

## Abstract

Objectives: The purpose of this study is to confirm that ANO1 correlates with occurrence and metastasis of OSCC. Study Design: Immunohistochemistry was used to detect the expression of ANO1 in 160 specimens of OSCC and normal tissues. Lentiviral silencing ANO1 was used in scc-25 cell line to study the cell migration and cell detachment. Results: Immunohistochemical staining revealed that ANO1 was expressed in a large majority (132 out of 160, 82.5%) of OSCC specimens and that the rate of ANO1 expression in OSCC was significantly higher than that of normal tissue (P<0.05); The rate of ANO1 expression was higher in metastatic tumors than in non-metastatic tumors, and the difference was significant (P<0.05). The results of cell migration assay showed that the percentage of cells through the membrane was 26.61 ±0.81 in assay group, and 54.26 ±3.74 in control group, respectively (t=-16.22,P<0.0001). The results of cell detachment assay showed that the percentage of cells detachment was 37.42 ±0.90 in assay group, and 87.38 ±1.59 in control group, respectively (t=-62.34, P<0.0001). The results of wound healing assay showed the assay group had a reduced migration rate compared with the control group in 32 h (F=1038.78, P<0.0001). Wound closure was no significantly different between the assay and control cells when DIDS was used in wound healing assay (F=4.61,P>0.05). Conclusions: Our study shows that abnormal expression of ANO1 correlated with the occurrence and metastasis of OSCC in clinical specimens and that silencing ANO1 greatly reduced migration ability of scc-25 cells. Calcium activated chloride channel activity of ANO1 promoted the cell migration. Thus, ANO1 could represent a new diagnostic biomarker and a potentially important therapeutic target of OSCC.

** Key words:**Oral squamous cell carcinoma, chloride channel, metastasis.

## Introduction

Oral squamous cell carcinoma (OSCC) encompasses at least 90% of all oral malignancies, and exhibits frequent local/regional invasion (1). The area is rich in vascular and lymphatic vessels, there is a high propensity for oral cancer to metastasis to the other sites of body. Despite multiple modalities of treatment, such as surgery, radiation, and chemotherapy, oral cancer continued to have one of the lowest 5-year survival rates and thus has been an important research topic for surgeons (2). Improvement in patient survival requires better understanding of tumor occurrence and metastasis, and needs an effective biomarker for the occurrence and metastasis of oral cancer, so that these aggressive tumors can be detected at the earlier stages in the disease development process and targeted therapeutic interventions for this type of cancer can be developed.

According to the results of other studies (3,4), amplifications, abnormal expression and activation of chloride channels have been known to correlate with tumor occurrence and metastasis in several types of cancer cells. For example, glioma cells have strong invasiveness. Chloride channels’ activation was detected in the development and progression process of glioma cells. Recent studies have shown that the upregulation of chloride channels in glioma cell membrane enhanced the ability of its migration (5,6). Chloride channels were activated when glioma cells migrated. The blockers of chloride channels could prevent the migration of glioma cells. Thus, the chloride channels also play an important role in the process of cell migration (7).

Anoctamin-1 (ANO1), also known as oral cancer overexpressed 2 (ORAOV2), is one of the chloride channel proteins in humans and is encoded by the ANO1 gene located on 11q13 (8), a chromosomal region that is frequently amplified in different types of human carcinomas (9,10). ANO1 has recently been identified as a Ca2+ -activated chloride channel (11-13). There is a well recognized link between expression of chloride channels and cancer occurrence and metastasis (14,15). However, a detailed analysis of ANO1 expression in oral squamous cell carcinoma (OSCC) and its functions is missing. It is thus still unclear how ANO1 contributes to malignancy in OSCC. The purpose of this study is to determine whether abnormal expression of ANO1 correlates with occurrence and metastasis of OSCC. Thus, we analyzed the expression of ANO1 in OSCC samples and defined its functions in human oral cancer cell line.

## Material and Methods

-Patients and samples

Histological studies were made of 160 patients with primary OSCC at the Department of Oral and Maxillofacial Surgery, the First Affiliated Hospital of Chongqing Medical University, between January of 2000 and December of 2011. None of the patients had been treated previously with chemotherapy or radiotherapy. All the participated patients were informed and agreed with this study. They were staged according to the UICC TNM Classification of Malignant Tumors (16). A fragment of primary tumour and a fragment of normal mucosal close to tumour were removed, fixed in 6% buffered formaldehyde and embedded in paraffin for immunohistochemical analysis. General characteristics of the patients involved in this study are presented in  Table 1.

**Table 1:**
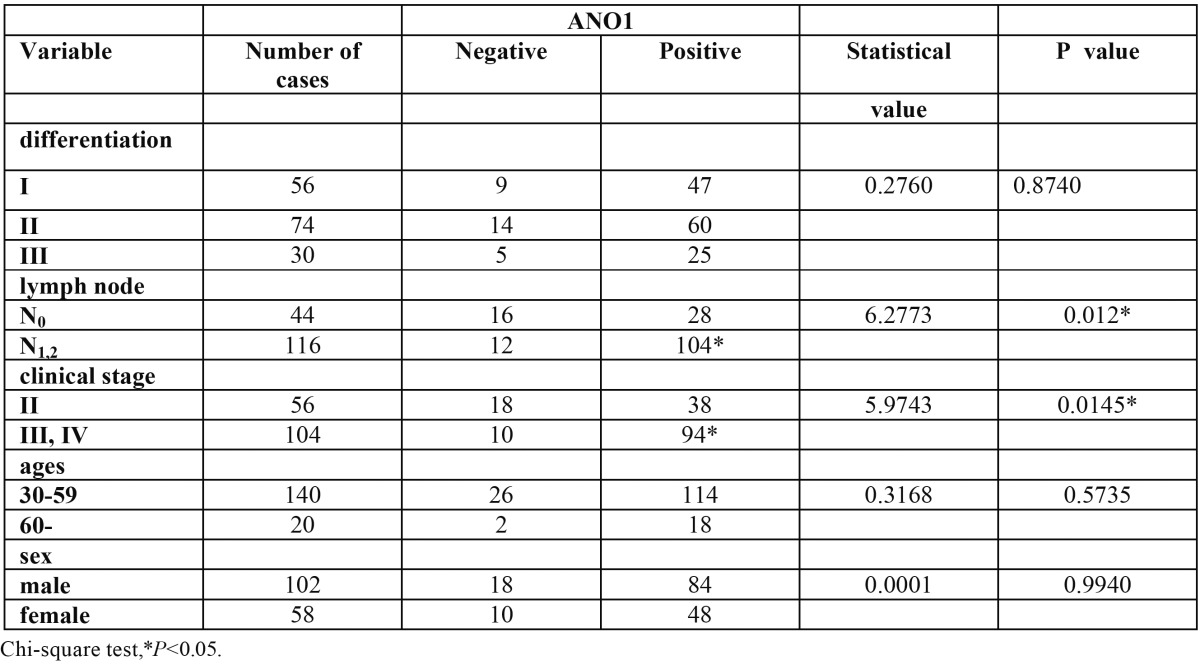
The relative of the expression of ANO1 and pathogenic and clinic parameters of OSCC patients.

-Immunohistochemical Analysis

Paraffin-embedded specimens were sectioned and pretreated in a microwave for 5 minutes for antigen retrieval. Incubation with primary antibody (rabbit polyclonal anti-ANO1 1/500 Sigma) was carried out at 4°C overnight. Peroxidase-labeled anti-rabbit immunoglobulin was used as secondary antibody. Streptavidia/Pemxida kit (ZYMED,USA) was used for revelation. The appearance of brown staining in the cell was considered positive for this protein.

-Cell culture

Tongue cancer accounts for a majority of the cases of oral and maxillofacial malignancies, so tongue cancer cell line (scc-25) was used in this study. Human cell line of scc-25 (Center Laboratory of Chongqing Medical University) were grown in modified Eagle’s medium (Sigma, USA), supplemented with 10% fetal calf serum, 1 mM Sodium pyruvate, 40µg/ml gentamycin. (Gibco, USA) at a temperature of 37°C in an environment containing 5% CO2.

Lentiviral-mediated siRNA targeted against ANO1

Three shRNA sequences targeting ANO1 (GeneBank accession NR_030691) and a negative sequence for control were designed and approved by BLAST software. The four sequences were synthesized as follows: negative control shRNA, 5’-CATGGAGTGGCACGTAGGT-3’; ANO1-shRNA 1: 5’-TTACGTGGCGTTCTTCAAA-3’; ANO1-shRNA 2: 5’-GCACGATTGTCTATGAGAT-3’; ANO1-shRNA 3: 5’-GAATCATTGTCTTCCTGTATT-3’. These shRNAs were cloned into pTY linkers (Lentiviral vectors). shRNA lentiviral vector was transiently transfected into HEK-293T cells via calcium phosphate-mediated transient transfection. Lentivirus was transfected into scc-25 cells.

-Reverse Transcription-Polymerase Chain Reaction (RT-PCR) 

Total RNA was extracted with GeneElute mammalian total RNA miniprep kit (Sigma), verified by agarose gel electrophoresis for integrity, reverse transcribed with Superscript II (RTase SC, Life Technologies) and oligodT primer (Sigma), and amplified by PCR using a LightCycler and LC Fast start DNA master SYBR green I kit (Roche Diagnostics). The primers were designed by primer3 software(http://frodo.wi.mit.edu/cgi-bin/primer3/primer3_www.cgi). The primer sequences for AN01 were:ANO1 forward primer:5’-CTCCTGGACGAGGTGGTATGG-3’ and reverse primer: 5’-GAACGCCACGTAAAAGATGG-3’; The primers for the ubiquitous gene ribosomal protein P0 (RPLP0) were: forward primer: 5’-GAAGGCTGTGGTGCTGATGG-3’ and reverse primer:5’-CCGGATATGAGGCAGCAGTT-3’. The specificity of the PRC amplified products was verified by agarose gel electrophoresis and expression levels of AN01 were normalized with RPLP0.

-Western blot analysis

Cells were lysed in 50ul lysis buffer(1 mM dithiothreitol, 0.125 mM EDTA, 5% glycerol, 1 mM phenylmethylsulfonylﬂuoride, 1 lg/ml leupeptin, 1 lg/mlpepstatin, 1 lg/ml aprotinin, 1% Triton X-100 in 12.5 mMTris–HCl buffer, pH 7.0) , and heat denatured. Proteins were separated by 10% sodium dodecyl sulphate-polyacrylamide gel electrophoresis (SDS–PAGE) and transferred to nitrocellulose membrane, blocked by 5% dry milk, and the membranes were incubated with anti-ANO1 (1/1000) and anti-TBP (1/2000,Sigma,USA) antibodies. Following incubation with horseradish peroxidase-conjugated secondary antibody and the membranes were developed with the enhanced chemiluminescence kit (Pierce).

-Cell Migration Assay

Transwell chambers with 6.5 mm polyvinyl/pyrrolidone-free polycarbonate filters of 8 μm pore size were used. Cells (4×105 mL−1) were suspended in 100 μL of serum-free medium in the upper transwell chamber; 10% FCS-containing medium was placed in the lower chamber. After 4 h of incubation at 37°C,cells on the upper surface of the filter were completely wiped away with a cotton swab, and the lower surface of the filter was fixed in methanol, stained with Giemsa and counted under a microscope.

-Cell detachment assay 

To measure detachment, cells were plated at a density of 4×104 mL−1 in 500μL of medium in 24-well plates (Integrated BioDiagnostics, Germany) and incubated for 24 h at 37°C. A stock trypsin-EDTA solution (0.05%) was diluted to 50% of the stock concentration. The cells were washed with phosphate-buffered saline (PBS) and incubated in diluted trypsin for 10 min at 37°C. All detached cells were collected following one wash with PBS. The detached cells and remaining cells were counted.

-In vitro wound healing assay

Cells were plated in duplicate in 24-well plates, grown to confluence, and scraped with sterile 200μL disposable plastic pipette tips and washed with PBS. Cells were incubated with serum free medium containing 5 μM Aphidicolin (Sigma) to inhibit cell proliferation. The wound healing procedure was observed by microscopy, and images were collected. The distance between the wound edges was measured using Adobe Photoshop CS2. Wound closure was presented as percentage of reduction of the freshly wounded area. To study the effects of 4-4′-Diisothiocyanatostilbene-2,2′-disulfonic acid disodium salt (DIDS), cells were incu-bated with 100μM DIDS ( dissolved in DMSO 0.1%) and photographed after 0, 8, 24 and 32 h.

-Statistical analysis

Statistical significance was assessed using Chi-square test, Student’s t-test and ANOVA. A P value < 0.05 was considered to be statistical significance.

## Results

-Expression of ANO1 in specimens of OSCC 

**Figure 1:**
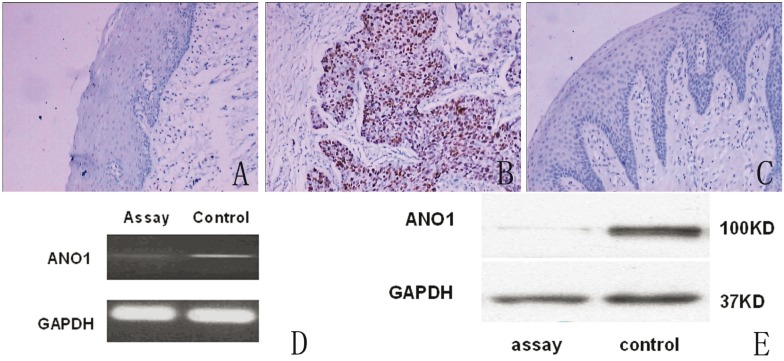
Expression of ANO1 in clinical specimens and the scc-25 cells. A: ANO1 is negative in oral normol tissure by immunohistochemical staining SP×200; B: ANO1 is positive in oral cancer tissure by immunohistochemical staining SP×200; C: ANO1 is negative in oral cancer tissure by immunohistochemical staining SP×200; D: The RT- PCR analysis of ANO1 mRNA levels in scc-25 cells after infection with lentivirus,the expression levels of ANO1 mRNA were (0.14±0.01) in assay group,(0.32±0.02) in control group, (Student’s t-test, t=-24.55, *P*<0.0001); E: The Western blots analysis of ANO1 protein expression in scc-25 cells after infection with lentivirus,the protein expression levels of ANO1 were(0.22±0.08) in assay group,(1.16±0.09)in control group , (t=-19.66, *P*<0.0001).

For immunohistochemical analysis, staining in all specimens was restricted to the tumor cells, with no staining of the stroma. No staining was detected in normal mucosal. Protein expression of ANO1 was detected in 132 (82.5%) of 160 cancer tissues. The rate of ANO1 expression was higher in metastatic tumors than in non-metastatic tumors, and the difference was statistically significant (Chi-square test, *P*<0.05). The rate of ANO1 expression increased in accordance to clinical stage (*P*<0.05). No significant differences between the rate of ANO1 expression and differentiation, sex and age were observed (*P*>0.05) (Fig. 1,  Table 1).

-Silencing efficacy of ANO1 with shRNA lentivirus

As assay group, the stable silencing of ANO1 scc-25 cell line was successfully built with ANO1- shRNA1 lentivirus. The control group of scc-25 cell line was successfully built with negative control shRNA lentivirus. The silencing efficacy of ANO1 shRNA lentivirus at mRNA and protein levels in scc-25 cells was determined by RT-PCR and Western blot, respectively. The results of RT-PCR showed that the expression levels of ANO1 mRNA were 0.14±0.01 in assay group and 0.32±0.02 in control group, respectively (Fig. 1). The ANO1 mRNA level in shRNA1 lentivirus-cells was reduced to 43.75% (a 2.29 fold reduction) of that in the control group. The difference in ANO1 mRNA levels between the assay and control group was statistically significant (Student’s t-test, t=-24.55, *P*<0.0001); and Western blot revealed that the protein expression levels of ANO1 were 0.22±0.08 in assay group, and 1.16±0.09 in control group, respectively (Fig. 1). The protein level of ANO1 in the assayed group was reduced to 18.97% (a 5.27 fold reduction) of that of the control group. The difference in ANO1 protein levels between the assay and control group was statistically significant (t=-19.66, *P*<0.0001). The results of both RT-PCR and Western blot demonstrated that lentiviral vectors were effective for ANO1 silencing.

-Effects of silencing ANO1 on cell migration and detachment 

**Figure 2:**
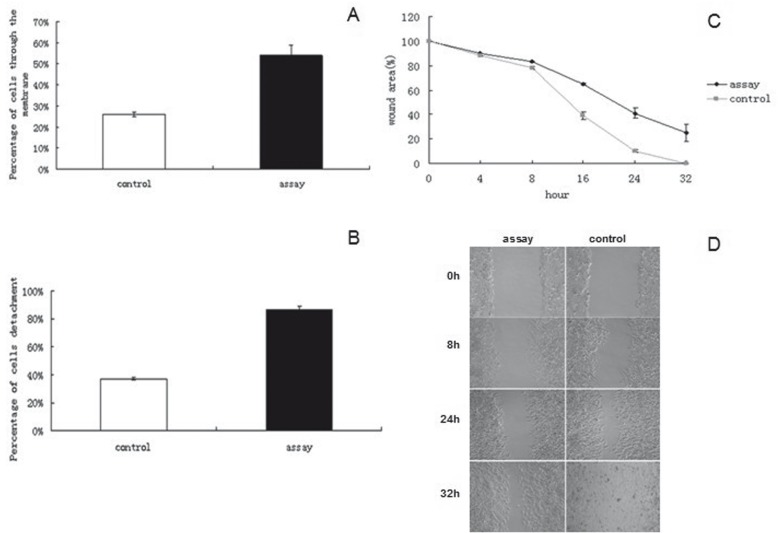
Effects of silencing ANO1 on cell migration and detachment. A: Transwell chambers assay was used to examine the effect of silencing ANO1 on cell migration. After incubation for 4 h, the percentage of cells through the membrane was (26.61 ±0.81) in assay group, (54.26 ±3.74) in control group, (t=-16.22, *P*<0.0001); B: Cell detachment assay was performed to examine the effect of silencing ANO1 on cell detachment. The results showed that the percentage of cells detachment was (37.42 ±0.90) in assay group, (87.38 ±1.59) in control group, (t=-62.34, *P*<0.0001); C and D: In vitro wound healing assay was performed to further investigate the effect of silencing ANO1 on cell migration. The results showed that the cells transfected with ANO1- shRNA1 lentivirus had a extended wound closure time compared with the control group in 32 h (ANOVA, F=1038.78, *P*<0.0001).

The effect of silencing ANO1 on cell migration was examined with Transwell chambers assay. After incubation for 4 h, the percentage of cells through the membrane was 26.61 ±0.81 in assay group, and 54.26 ±3.74 in control group, respectively (t=-16.22, *P*<0.0001) (Fig. 2) and the cell migration rate in assay group was reduced by 2.04 fold of that in the control group. The results of this experiment suggested that silencing ANO1 expression reduced migration ability of scc-25 cells. To examine the effect of silencing ANO1 on cell detachment, cell detachment assay was performed. The results showed that the percentage of cells detachment was 37.42 ±0.90 in assay group and 87.38 ±1.59 in control group, respectively (t=-62.34, *P*<0.0001) (Fig. 2) and the cell detachment rate in assay group was reduced by 2.34 fold of that in the control group. To further investigate the effect of silencing ANO1 on cell migration, in vitro wound healing assay was performed. The results showed that the cells transfected with ANO1- shRNA1 lentivirus had an extended wound closure time as compared with that in the control group in 32 h (ANOVA, F=1038.78, *P*<0.0001) (Fig. 2). Silencing ANO1 in human scc-25 cells led to reduced cell migration and detachment to a greater extent than that in control cells, implying that ANO1 knockdown may preferentially inhibit metastasis of human scc-25 cells.

-DIDS decrease scc-25 cell migration in control cells

**Figure 3:**
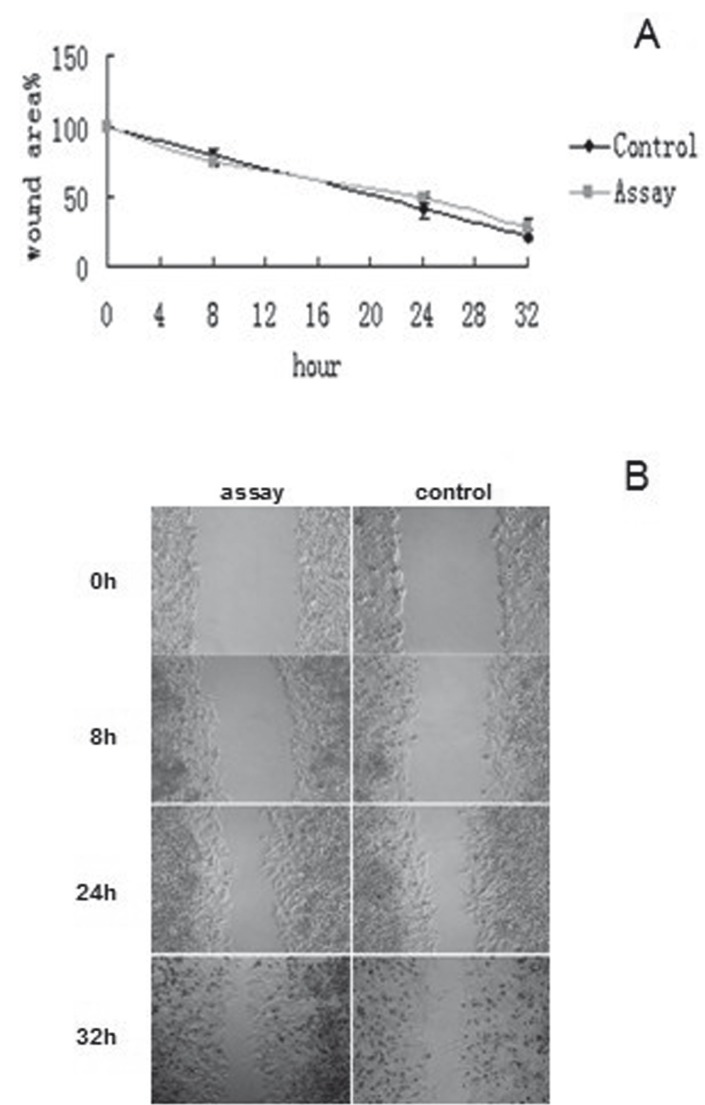
DIDS decreases scc-25 cell migration in control cells. A,B: To investigate whether calcium activated chloride channel activity of ANO1 promote the cell migration, the inhibitor (DIDS) was used in vitro wound healing assay. The results showed that wound closure were no signifcantly different between the assay and control cells (F=4.61, *P*>0.05)

To investigate whether calcium activated chloride channel activity of ANO1 promotes the cell migration, DIDS, an inhibitor of anion exchange channel, was used in in vitro wound healing assay. The results showed that DIDS delayed wound closure in the control cells, since the expression of ANO1 was not knockdown. The lack of an effect on assay cells is expected because of si-lencing ANO1. So wound closure was no significantly different between the assay and control cells (F=4.61, P>0.05) (Fig. 3), which showed that ANO1 channel activity participated in the scc-25 cell migration.

## Discussion

ANO1 belongs to a novel family of membrane chloride proteins with cytosolic amino- and carboxyl termini facing the cytoplasm (17). ANO1 has been suggested to drive 11q13 amplification by providing growth or metastatic advantage to tumours. Several studies have indicated that ANO1 may be of clinical value as a diagnostic and prognostic biomarker and therapeutic target of tumours (18,19).

Our study indicated that ANO1 could be a marker of occurrence and metastasis in OSCC.

In this study, we reported, for the first time, a relatively detailed analysis of ANO1 expression in OSCC. First, we performed the analysis of the expression of ANO1 in 160 patient samples of primary OSCC with immunohistochemical assay. We have shown that ANO1 expression was found in a large majority (132 out 160, 82.5%) of oral cancer tissue, but not in normal tissue and its expression level was higher in metastatic tumors than in non-metastatic tumors. Furthermore, we also observed that ANO1 ex-pression strongly correlated with the increasing clinical stage of OSCC patients. These results are in accordance with those re-ported in other studies (18,20). A correlation between the expression of ANO1 and various types of cancers and diseases including urinary bladder cancer, breast cancer, head and neck squamous cell carcinoma and cystic fibrosis has been reported (8,20,21). ANO1 is rarely expressed in corresponding normal tissues. Our results imply that ANO1 correlates with the occurrence (22) and the metastasis of OSCC, and that ANO1 could act as an independent marker of occurrence and metastasis for OSCC.

To explore the cellular function of ANO1, we conducted a series of experiments with scc-25 cell line. First, we knocked down ANO1 expression in scc-25 cell line with shRNA lentivirus and examined the effects knocking down ANO1 expression on cell migration and detachment with cell migration assay, detachment assay and in vitro wound healing assay with silencing ANO1 scc-25 cell line. We detected significantly reduced cellular migration and detachment in scc-25 cell caused by silencing ANO1. Detachment and migration of cells are essential steps for metastasis of tumor cells, to regional and distant sites which threatens patients’ lives. By reducing metastasis of cancer cells could be one of the effective ways to save many patients lives from cancers. Our study showed that silencing ANO1 might be useful to prevent tumor metastasis at least in OSCC.

ANO1 is a Ca2+-activated chloride channel (11-13). The functions of Ca2+-activated chloride channel include governing cellular ion fluxes and regulating cell volume (23). There is substantial evidence that cell migration is facilitated by the function of chlo-ride channels (3,6,24). The process of cell migration comprises cell swelling at the leading edge and subsequent cell shrinkage at the rear part of the cell (25). The chloride channels are involved in the changes in cell migration (26). When cells migrate, they need to change their volume and also require the involvement of cytoskeleton. Thus, being a Ca2+-activated chloride, ANO1 might be involved in the control of cell migration by regulating the cell volume. Indeed, Ruiz et al. found that ANO1 regulated cell volume and causes cell migration (20). Perez-Cornejo found that ANO1 might play a role in organization of the actin cy-toskeleton (27). Therefore, it is likely that ANO1 accelerates the migration of cancer cells by regulating the coordinated changes in cell volume and organization of cytoskeleton.

It is quite reasonable that if ANO1 is involved in cell migration, then inhibition of its activity should block or reduce cell migration. Indeed, it has been reported that blocking chloride channel inhibits migration of nasopharyngeal carcinoma cells (28), and that inhibition of ANO1 suppressed the migration of prostate carcinoma cells in vitro (18). Thus, we used DIDS, an inhibitor of anion exchange channel, to examine its effects on wound closure using in vitro wound healing assay. Our results showed that DIDS delayed wound closure in scc-25 cell line. The results also support the concept of chloride channel being an essential factor that determines abilities of the cells to migrate and to metastasize (29,30). We reasoned that chloride channel activity of ANO1 takes part in cell migration which facilitates the emergence of metastasis in OSCC and in other cancers as well.

In addition to its role in regulation of cell migration and metastasis, ANO1 may be also involved in other cellular processes. Duvvuri et al. found that ANO1 could improve cancer cell proliferation and increased activation of extracellular signal-regulated kinase (ERK) at the same time and silencing ERK could abrogate the growth effect of ANO1 (22). Also, ANO1 induced prolifera-tion by mitogen-activated protein kinase (MAPK) activation (22).

Taking these lines of evidence together, it is reasonable to conclude that abnormal expression and activation of ANO1 correlates with the occurrence, progression and metastasis of cancer and that ANO1 could be an important target of therapeutic drugs. However, since the role of ANO1 in cell migration and detachment is determined in vitro studies, whether or not this finding can be transferable to the in vivo situation requires to be further verified. Thus, further experimental work will be required to define the precise roles of ANO1 on OSCC metastasis in vivo for the development of small-molecule inhibitors against ANO1.
